# Taking Comfort in Life

**Published:** 1881-07

**Authors:** 


### TAKING COMFORT IN LIFE.

Sooner or later, friends, the time for folded hands will come to us all. Whether or not we cease from hurry and worry now, we shall one day shut our eyes upon it and lie still, untroubled by the stir and fret of things about us. Why not take comfort as we go on? You, proud mother of a beautiful, active boy, of what use will it be to you to remember how exquisitely fine was his raiment, how daintily spread his bed, and how costly and profuse his toys? What the child needs is mothering, brooding, tender resting on your heart, and he needs it every step of the way from babyhood to manhood. Take the comfort of your opportunities, never mind though the dress be coarse, and the food plain, and the playthings few, but answer the questions, tell the stories, spare the half hour at bed time, and be merry and gay, confidential and sympathetic with your boys. And you, whose graceful young daughter is just blushing out into the bloom and freshness of a wondrously fair womanliness, do not be so occupied with your ambition for her own advancement in life that you let her ways and your own fall apart. Why does she visit here and there, and receive visitors from this and that home, and you scarcely know the people by sight? You are losing precious hours, and the comfort you ought to take is flying fast away on these wings of time that are never overtaken.

### ICEBERGS.

Pyer estimated that in an iceberg 200 feet above the water a total of 600 to 800 feet may, as a rule, be inferred. Sir John Ross estimated the highest iceberg he had seen at 51 feet; Koldmay found one of 220 feet; Weyprecht and Payer one of 200 feet; Baffin another of 240 feet; Payer, 258 feet; Kane, 300 feet; Back, 300 feet; and as about seven-eights of the berg are under water, the curious spectacle is witnessed of these great masses plowing their way against a rapid current loaded with heavy pack ice, and in the very teeth of a strong gale. The reason of this peculiar sight is that the deep current, which directs the drift of the iceberg, is far more powerful than the shallow surface one which is visible.—*Lieut Schwatka.*

The new Czar leads a very simple life. He rises early and takes a long walk, then breakfasts with his family, after which he goes down cellar and covers himself up in a coal-bin for the balance of the day to keep out of the way of the Nihilists.

